# Bevacizumab in recurrent glioblastoma: does dose matter? Our monocentric and comparative experience

**DOI:** 10.1007/s11060-025-04992-4

**Published:** 2025-03-10

**Authors:** Giulia Cerretti, Alberto Bosio, Giovanni Librizzi, Giovanna Pintacuda, Mario Caccese, Alessandro Salvalaggio, Marco Zoccarato, Alessandro Parisi, Marta Padovan, Marta Maccari, Francesco Cavallin, Luisa Bellu, Francesco Pasqualetti, Tamara Ius, Luca Denaro, Francesco Volpin, Marina Coppola, Sara Lonardi, Giuseppe Lombardi

**Affiliations:** 1 Medical Oncology 1, Veneto Institute of Oncology IOV-IRCCS, Via Gattamelata n°64, 35128 Padua, Italy; 2 Department of Surgery, Oncology and Gastroenterology, University of Padua, Via Gattamelata n°64, 35128 Padua, Italy; 3 Neuroradiology, Department of Neurosciences, University of Padova, Padova, Italy; 4 Padova Neuroscience Center (PNC), University of Padova, Padova, Italy; 5 Radiology Unit, Veneto Institute of Oncology IOV-IRCCS, Padova, Italy; 6 Department of Neuroscience, University of Padova, Padova, Italy; 7 Neurology Unit O.S.A, Azienda Ospedale-Università di Padova, Padova, Italy; 8 Radiotherapy Unit, IOV-IRCCS Veneto Institute of Oncology, Padova, Italy; 9 Independent Statistician, Solagna, Italy; 10 Academic Neurosurgery, Department of Neurosciences, University of Padova, Padova, Italy; 11 Division of Neurosurgery, Azienda Ospedaliera Università di Padova, Padova, Italy; 12 Pharmacy Unit, Veneto Institute of Oncology IOV-IRCCS, Padova, Italy

**Keywords:** Bevacizumab, Recurrent glioblastoma, Bevacizumab dose, Toxicity, Antiangiogenesis, Glioblastoma

## Abstract

**Purpose:**

Bevacizumab is an anti-angiogenetic treatment that can be used in patients with recurrent glioblastoma, but there are limited and controversial data on the optimal dose and schedule, associated toxicities and survival benefits of different doses.

**Methods:**

A retrospective analysis of patients with recurrent *IDH*wt glioblastoma treated with bevacizumab at the Veneto Institute of Oncology was performed. Patients received bevacizumab in 2 different schedules (5 mg/kg or 10 mg/kg q2w), as monotherapy or in combination with chemotherapy.

**Results:**

81 patients were analyzed, 33 received bevacizumab 5 mg/Kg, 48 received bevacizumab 10 mg/Kg. Median PFS was 4 months in both patients treated with 5 mg/kg and those treated with 10 mg/kg (p-value=0.08), median OS was 5 months in patients treated with 5 mg/kg and 7 months in those treated with 10 mg/kg (p-value=0.10). There was no difference in the use of steroid therapy between the two groups. The incidence of adverse events was not statistically different.

**Conclusions:**

There was no statistically significant difference in survival, PFS, response, toxicity and steroid reduction between the two different doses. These results may support the use of lower doses of the drug with comparable benefit for patients and with additional advantage in terms of health care costs.

**Supplementary Information:**

The online version contains supplementary material available at 10.1007/s11060-025-04992-4.

## Background

Glioblastoma (GBM) is the most common primary malignant brain tumor in adults [1]. The prognosis is poor with a median survival of 15–18 months and a 5-year survival inferior to 7%. Standard treatment involves surgical resection followed by radiation and chemotherapy [1]. Available therapeutic strategies, which involve the use of nitrosureas, antiangiogenetic treatments and alkylating agents, are few and have demonstrated limited benefit so far [2]. Since malignant gliomas are very vascular tumors in which angiogenesis plays a critical pathologic role, many studies on inhibiting angiogenesis have been conducted in the past years [3].

Brain tumor angiogenesis, which is closely associated with brain tumor progression, is mediated through the action of many angiogenic factors including vascular endothelial growth factor (VEGF), basic fibroblast growth factor (bFGF), hepatocyte growth factor (HGF), platelet-derived growth factor (PDGF), and TGF-β, MMPs, and angiopoietins (Angs) [4]. 

Bevacizumab is a recombinant, humanized monoclonal antibody that binds to VEGF-A, thereby inhibiting VEGFR-mediated cell signaling. It is generally administered every 2–3 weeks [3, 5, 6].

The role of bevacizumab in high grade gliomas has been extensively studied, both in newly diagnosed glioblastoma and in the recurrent setting [7].

In 2009, the US Food and Drug Administration (FDA) granted accelerated approval for bevacizumab monotherapy in patients with recurrent glioblastoma based on the neuroradiological response demonstrated by two phase II trials [7–9]. On the contrary, the European Medicines Agency (EMA) did not grant approval due to absence of a control group, inadequate response criteria and difficulty in interpreting OS and PFS outcomes [10].

Over the years, some trials investigated the role of bevacizumab both as monotherapy or combined with cytotoxic agents using different dosages and schedules of bevacizumab [8, 11–18].

Although the use of bevacizumab is well established in clinical practice, there is currently no consensus on its dosage and schedule, as few dose-response studies have been performed recently [19, 20]. The most common dosage for bevacizumab is 10 mg/kg every two weeks and it was based on protocols for colon-rectal cancer [20]. However, other schedules such as 5 mg/Kg/week or 7.5 mg/Kg every 3 weeks have also been evaluated in terms of efficacy and safety [19, 21, 22], either in combination with chemotherapy or alone [19].

In this paper, we retrospectively describe our large mono-institutional experience with bevacizumab in recurrent glioblastoma patients analyzing two different schedules of bevacizumab in terms of survival outcomes, response and safety.

## Materials and methods

We retrospectively evaluated all patients treated with bevacizumab for a recurrent isocitrate dehydrogenase (*IDH*) wild-type glioblastoma at the Veneto Institute of Oncology (Padova, Italy) between May 2013 and February 2022. Patients were treated with two different doses of bevacizumab: 5 mg/Kg (low dose- LD) or 10 mg/Kg (high-dose- HD) every 2 weeks; the choice was at the clinician’s discretion. Eligibility criteria included (i) treatment with bevacizumab after relapse to at least first line therapy (ii) availability of histological, clinical and radiological data at the assessment time-points. Bevacizumab could be administered in LD or HD, in combination or as monotherapy at the physician’s discretion. Bevacizumab was prescribed as an off label therapy. All patients provided a written informed consent for the collection and use of their anonymized data for scientific purposes. Protocol has been approved by local ethical committee (EC n.7/2024).

The primary endpoint was the overall survival (OS), calculated from the date of the first administered dose of bevacizumab to the date of death or last follow-up. The secondary endpoint included neuroradiologic response according to Response Assessment in Neuro-Oncology (RANO) criteria [23], the treatment toxicity according to the Common Terminology Criteria for Adverse Events (CTCAE v5.0 [24]), and the progression-free survival (PFS), calculated from the date of the first administered dose of bevacizumab to the date of disease progression according to RANO criteria or last follow-up. As in standard clinical practice, the magnetic resonance imaging (MRI) assessments were performed approximately every two months or as clinically indicated.

All data were extracted from the electronic database of the Veneto Institute of Oncology and collected in an anonymized dedicated database. Data collection included demographics, tumor characteristics, treatment data (including treatment-related adverse events), and follow-up information. Methylation status of the O6-methylguanine DNA methyltransferase (*MGMT*) promoter was determined by methylation-specific polymerase chain reaction (PCR) or DNA pyrosequencing (7% cut-off for methylation). *IDH* mutation status was analyzed by immunohistochemistry or PCR in the case of patients ≤ 55 years. Pathological and molecular analysis confirmed that all available tissue samples from primary or recurrent tumors represented IDH wild-type GBM according to the World Health Organization classification of central nervous system tumors (WHO 2021) [25].

Statistical analysis was performed using R 4.4 (R Foundation for Statistical Computing, Vienna, Austria) [26]. Categorical data were summarized as number and percentage, and continuous data as median and interquartile range (IQR). Comparisons between the two groups were performed using the Chi-square test or the Fisher’s exact test (categorical data), or the Mann-Whitney test (continuous data). Survival curves were calculated using the Kaplan-Meier method and compared between the groups using the log-rank test. Cox regression models were estimated to assess the effect of bevacizumab dosage (5 vs. 10 mg/Kg) on OS and PFS, adjusting for major clinical confounding factors including age, prior lines of therapy, Eastern Cooperative Oncology Group (ECOG) performance status, and *MGMT* status. The administration of bevacizumab in association or as monotherapy could not be included in the models because only one patient in 5 mg/Kg group was treated in association. All tests were 2-sided and a p-value of less than 0.05 was considered statistically significant.

## Results

The analysis included 81 patients. The dose was 5 mg/Kg in 33 patients and 10 mg/Kg in 48 patients. Patient characteristics are summarized in Table 1. No patients received re-irradiation. Baseline characteristics were not statistically different between the two groups, except for older age in patients treated with Bevacizumab 5 mg/kg (*p* = 0.005), whose treatment was administered more frequently as monotherapy (97% vs. 77% in patients treated with 5 mg/kg bevacizumab, *p* = 0.03).

**Table 1:** Baseline characteristics in patients with recurrent glioblastoma who were treated with bevacizumab at a dosage of 5 mg/kg or 10 mg/kg administered every two weeks

Baseline characteristics	Patients treated with 5 mg/Kg bevacizumab (*n* = 33)	Patients treated with 10 mg/Kg bevacizumab (*n* = 48)	*p*-value
Males	18 (54%)	34 (71%)	0.21
Age, years	57 (49–65)	50 (41–60)	0.005
Methylated *MGMT*	15/30 (50%)	20/44 (45%)	0.88
Number of prior surgeries: 1 surgery 2 surgeries	24 (73%) 9 (27%)	27 (56%) 21 (44%)	0.20
ECOG performance status: 0–1 2–3	16 (48%) 17 (52%)	29 (60%) 19 (40%)	0.40
Prior lines of therapy 0–1 lines ≥ 2 lines	10 (30%) 23 (70%)	12 (25%) 36 (75%)	0.78
Bevacizumab administration: Monotherapy Association with chemotherapy ^a^	32 (97%) 1 (3%)	37 (77%) 11 (23%)	0.03

Data summarized as n (%) or median (IQR) ^a^

Bevacizumab 5 mg/Kg was administered in association with temozolomide (1 patient); bevacizumab 10 mg/Kg was administered in association with temozolomide (4 patients), irinotecan (2 patients), lomustine (2 patients), and fotemustine (3 patients)

**Fig. 1:**
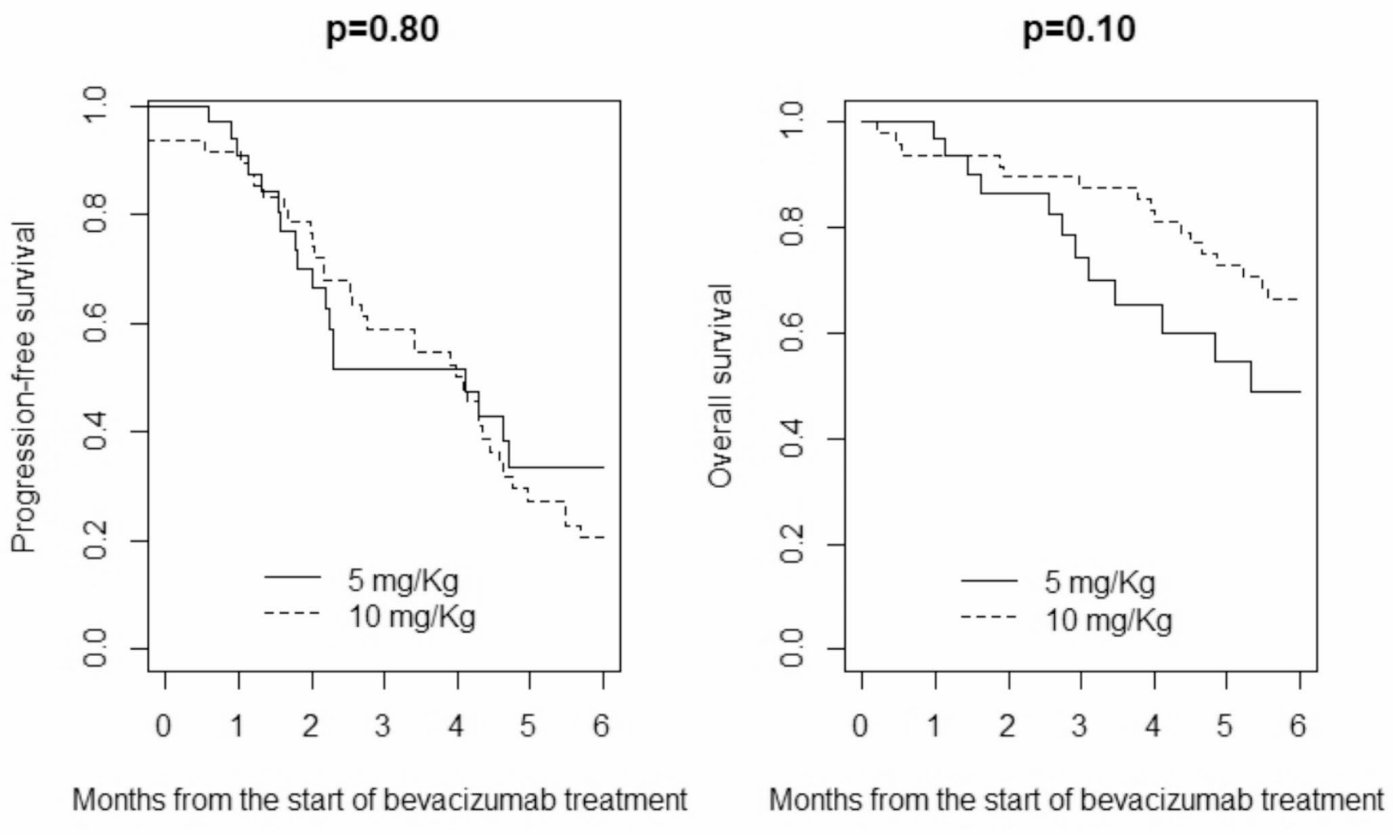
Progression-free survival (left) and overall survival (right) in patients treated for recurrent glioblastoma with 5 or 10 mg/Kg bevacizumab

The median follow-up from the start of bevacizumab treatment was 5 months (IQR 3–9). At the time of analysis, 62 patients (77%) had experienced a disease progression and 50 patients (62%) died. Median PFS was 4.0 months in both patients treated with 5 mg/Kg and in those who were treated with 10 mg/Kg. Six-month PFS was 33% in patients who were treated with 5 mg/Kg and 20% in patients treated with 10 mg/Kg (*p* = 0.80) (Fig. 1).

There was no statistically significant difference in overall survival between the two groups; median OS was 5 months in patients who were treated with 5 mg/Kg and 7 months in those who were treated with 10 mg/Kg. Six-month OS was 49% in patients who were treated with 5 mg/Kg and 66% in patients treated with 10 mg/Kg (*p* = 0.10) (Fig. 1).

In multivariable analysis (Table 2), bevacizumab dose was not associated with progression-free survival (*p* = 0.77) or overall survival (*p* = 0.32). Higher ECOG PS was associated with worse progression-free survival (hazard ratio 1.97, 95% confidence interval 1.15 to 3.39), whereas methylated *MGMT* was associated with improved progression-free survival (hazard ratio 0.43, 95% confidence interval 0.25 to 0.75) and improved overall survival (hazard ratio 0.49, 95% confidence interval 0.26 to 0.93).

**Table 2:** Multivariable analysis of progression-free survival and overall survival in recurrent glioblastoma patients treated with 5 or 10 mg/kg bevacizumab

Variable	Progression-free survival		Overall survival	
	Hazard ratio (95% confidence interval)	p-value	Hazard ratio (95% confidence interval)	p-value
Bevacizumab dose: 5 mg/Kg 10 mg/Kg	Reference 1.11 (0.62 to 1.97)	0.72	Reference 0.65 (0.33 to 1.30)	0.22
Age, years	1.00 (0.97 to 1.02)	0.75	0.98 (0.95 to 1.01)	0.31
Prior lines of therapy: 0–1 lines ≥ 2 lines	Reference 0.92 (0.50 to 1.67)	0.79	Reference 0.59 (0.30 to 1.16)	0.13
ECOG PS: 0–1 2–3	Reference 1.97 (1.15 to 3.39)	0.01	Reference 1.80 (0.98 to 3.33)	0.06
*MGMT*: Unmethylated Methylated	Reference 0.43 (0.25 to 0.75)	0.003	Reference 0.49 (0.26 to 0.93)	0.02

Response rates were not statistically different between patients treated with 5 mg/Kg or 10 mg/Kg of bevacizumab *(*Table 1S, *supplementary materials)*. Seven patients could not be assessed for response due to death, progressive disease in the first cycle, or failure to reach the first follow-up MRI scan. No complete responses were observed. Steroid therapy during bevacizumab administration is summarized in Table 3. Concomitant steroid therapy was not statistically different between patients who were treated with 5 mg/Kg or 10 mg/Kg bevacizumab at baseline, 3 and 6 months in patients without disease progression.

**Table 3:** Steroid therapy during bevacizumab administration

Timing	Steroid therapy	Patients treated with 5 mg/Kg bevacizumab (*n* = 33)	Patients treated 10 mg/Kg bevacizumab (*n* = 48)	*p*-value
Baseline	Steroid therapy	28 (85%)	21/47 (68%)	0.15
	Dexamethasone mg equivalents per patient	4 (2–6)	3 (2–5)	0.25
3 months	Steroid therapy	11/17 (65%)	18/32 (56%)	0.79
	Dexamethasone mg equivalents	2 (1–4)	2 (1–3)	0.91
6 months	Steroid therapy	3/5 (60%)	8/12 (67%)	0.99
	Dexamethasone mg equivalents	2 (1–2)	4 (2–4)	-

Data summarized as n (%) or median (IQR)

Adverse events during bevacizumab administration are summarized in Table 4. There were no deaths or bevacizumab discontinuations due to toxicity. The incidence of adverse events was not statistically different between patients who were treated with 5 mg/Kg or 10 mg/Kg bevacizumab in terms of hemorrhage (*p* = 0.51), proteinuria (*p* = 0.81), hypertension (*p* = 0.72), or thromboembolism (*p* = 0.99).

The most common adverse event was hypertension in both LD and HD bevacizumab. Thromboembolic events were all grade I-II.

**Table 4:** CTCAE V.5.0 adverse events during bevacizumab administration

Toxicity	Patients treated with bevacizumab 5 mg/Kg (*n* = 33)		Patients treated with bevacizumab 10 mg/Kg (*n* = 48)	
	Grade I-II	Grade III	Grade I-II	Grade III
Leading to death	0 (0%)	0 (0%)	0 (0%)	0 (0%)
Leading to discontinuation of bevacizumab	0 (0%)	0 (0%)	0 (0%)	0 (0%)
Hemorrhage: Overall Intracranial	0 (0%) -	0 (0%) -	2 (4%) 0 (0%)	0 (0%) -
Proteinuria	2 (6%)	0 (0%)	5 (10%)	1 (2%)
Hypertension	15 (45%)	6 (18%)	18 (37%)	8 (16%)
Thromboembolism: Overall Venous Arterial	1 (3%) 0 (0%) 1 (3%)	0 (0%) - -	2 (4%) 1 (2%) 1 (2%)	0 (0%) - -

Data summarized as n (%)

## Discussion

The use of bevacizumab in recurrent glioblastoma has long been investigated, both as a single agent and in combination with other chemotherapeutic agents. After FDA approval in 2009, the 10 mg/Kg q2w schedule became the standard of care [27]; however, other schedules are commonly used and there is currently no clear indication. The literature also supports the use of different schedules and dosages of bevacizumab in the treatment of other CNS tumors, such as meningioma, as shown in the systematic review by Franke et al. [28]

According to the available literature bevacizumab in monotherapy has achieved a 6 m-PFS of 29-42.6%, a median PFS of 3–10 months and a median OS of 6.5–9.2 months *(*Table 2S, *supplementary materials).* Combination therapies showed a 6 m-PFS of 50.3%, a mPFS of 3.5-6 months and a mOS between 6.9 and 10.5 months *(*Table 3S, *supplementary materials).* Our survival data are consistent with previously available results. In this retrospective cohort of 81 recurrent *IDH*wt glioblastoma patients, two different schedules of bevacizumab were evaluated for survival, response rates, toxicity and concomitant steroid therapy. Bevacizumab was administered as an off-label treatment (both in combination therapy and as a single agent) in two schedules, a low-dose schedule (5 mg/Kg q2w) and a high-dose schedule (10 mg/Kg q2w); the choice of the different schedules was at the clinician’s discretion.

The baseline characteristics of the population were generally well balanced between the two groups; the groups differed in terms of age (patients receiving bevacizumab 10 mg/kg were slightly younger) and drug combination (patients were more likely to receive bevacizumab 5 mg/kg as monotherapy). Overall, it is important to note that the majority of patients (85%) received bevacizumab as monotherapy. The study did not show a meaningful difference in either mPFS or mOS. Similar results were found in the retrospective study by Gleeson et al., who found no difference in survival between higher and lower doses [29]. Wong et al. performed a meta-analysis including 548 malignant glioma patients treated with a bevacizumab-based therapy with different dosage regimens, distributed in 15 reports. No difference in outcome was found between 5 mg/Kg and 10-15 mg/Kg.

Blumenthal et al. retrospectively analyzed a cohort of recurrent glioblastoma patients treated with bevacizumab at 5 mg/Kg (cohort A) or 10 mg/Kg every 2 weeks (cohort B), combined or not combined with other systemic treatments, during a 7-year experience. In cohort A 30/87 (34%) of patients received bevacizumab as monotherapy and in cohort B 60/75 (80%) of patients received bevacizumab as monotherapy. In the subgroup of patients treated with bevacizumab as a single agent, mPFS was 3.1 months with bevacizumab 5 mg/Kg and 3.6 months with bevacizumab 10 mg/Kg was obtained, while mOS was 5.9 and 7.2 months respectively; no statistically significant difference was detected [20]. Such data, obtained in a subgroup of patients treated with bevacizumab alone, corroborate our results.

Very recently, Melhem et al., demonstrated that in recurrent glioblastoma patients treated with bevacizumab, the LD schedule was associated with a statistically significant improvement in both progression-free survival and overall survival as compared to the higher dose schedule of 10 mg/kg given every 2 weeks [30]. Indeed, a statistically significant difference in mPFS in LD and SD was seen (5.89ms and 3.22ms respectively) and also in mOS was (10.23ms vs. 6.28ms respectively). However, there are some differences in patient characteristics compared to our patients: 11.4% received re-irradiation vs. 0% in our study, about 80% received bevacizumab at first or second recurrence (vs. 54%), 36% had an ECOG PS ≥ 2 (vs. 46% in the present study). Only 3% received bevacizumab in combination with chemotherapy (vs. 13%).

In terms of neuroradiologic assessment according to RANO criteria, the DCR did not differ significantly between the different doses of bevacizumab: 43% with bevacizumab 5 mg/kg vs. 54% with bevacizumab 10 mg/kg; no complete responses were seen in either group, but there were more PRs in the LD group and more SDs in the HD group. To note, being a retrospective study, imaging evaluation was performed according to RANO criteria, which take into account both enhancing and nonenhancing tumor burden due to the occurrence of pseudoresponse in high-grade gliomas treated with antiangiogenic drugs such as bevacizumab. On the other hand, according to recently published RANO 2.0 criteria, nonenhancing disease evaluation is optional in patients undergoing such treatment, as the vast majority of patients has a concurrent progression of enhancing and nonenhancing disease [31, 32]. Therefore, we expect our results to be consistent with more recent assessment criteria.

No patient experienced AEs leading to death or AEs leading to treatment discontinuation.

There were no differences in serious adverse events between the two groups. Bevacizumab HD had 19% of bevacizumab-related grade ≥ 3 AEs versus 18% in LD, similar to previous studies [33]. No grade ≥ 3 thromboembolic events were recorded. Proteinuria and hypertension were present at comparable percentages across severity grades in LD and HD patients. Brain hemorrhage cases were 2, both ≤ grade 2 and both with HD bevacizumab. As expected, the most common toxicities in both groups were hypertension (grade ≥ 3 18.2% in the 5 mg/Kg and 16.7% in the 10 mg/Kg schedule) and proteinuria (grade ≥ 3 0% in the 5 mg/Kg and 2.1% in the 10 mg/Kg schedule). Both doses resulted in similar safety and toxicity rates in our study, comparable to previous data in the literature. Previous studies have produced controversial results regarding toxicity; Blumenthal et al. found a higher rate of AEs with bevacizumab HD, while Melhem et al. found higher toxicity in the LD group (although overall toxicity rates were low and the study is therefore underpowered to detect differences; in particular, no grade 3 hypertension and only 5.4% grade 1–2 hypertension were detected). Sirven-Villaros et al. reported that a reduced dose of bevacizumab (doses of 1 to 5 mg/kg) was better tolerated than routine bevacizumab (10 mg/kg), with fewer discontinuations due to toxicity [34].

According to previous data, the use of bevacizumab was associated with reduced dexamethasone mg equivalents in 23–58% of cases [11–13, 35]; conversely, our study did not show a significant different in steroid dose reduction between HD and LD. There is a trend toward a higher dexamethasone reduction between baseline and 3 months in the LD group, but it is possible that steroid dose reduction is independent of bevacizumab dose, and may be related to patient selection.

The value of the study lies in the large number of patients included and its real-world value. However, it has some limitations, such as the retrospective design, the fact that it is monocentric, and the fact that the schedule was chosen based on the clinician’s choice, allowing for possible bias.

Moreover, no data about anatomic location of GBM in relation to white matter tracts density was collected [36]. Furthermore, perfusion metrics could be useful in the prognostic stratification of recurrent glioblastomas treated with bevacizumab [37, 38], but were not available for most of our patients.

It is desirable that such results be confirmed in larger cohorts and prospective studies. In conclusion, this retrospective study confirms the role of bevacizumab as a useful treatment strategy in recurrent glioblastoma. Our data could encourage the use of a lower dosage of the drug, based on the lack of significant differences in survival outcomes, response rates and toxicity profiles. It should be recalled that in Europe (EMA) and consequently in Italy (AIFA) bevacizumab is not approved for the treatment of recurrent glioblastoma and is therefore administered as an off-label drug, financed by public health resources; the possibility to use effective lower doses of the drug would result in a significant benefit in terms of health care costs, as showed in a study by Gleeson et al. (2.4 M euros saving) [29]. 

It is in any case of the utmost importance to bear into mind the role of simultaneous or exclusive palliative care in this setting, based on the patients’ clinical caracteristics.

## Electronic supplementary material

Below is the link to the electronic supplementary material.

Supplementary Material 1

## Data Availability

No datasets for this retrospective study are not available.

## References

[1] Da LN (2017) High-grade gliomas. Continuum (Minneapolis Minn) 23(6). Neuro-oncology10.1212/CON.0000000000000554 10.1212/CON.0000000000000554 29200110

[2] Leone A, Colamaria A, Fochi NP et al (2022) Recurrent glioblastoma treatment: state of the Art and future perspectives in the precision medicine era. Biomedicines 10(8):1927. 10.3390/biomedicines10081927 36009473 10.3390/biomedicines10081927 PMC9405902

[3] Chamberlain MC, Raizer J (2009) Antiangiogenic therapy for high-grade gliomas. CNS Neurol Disord Drug Targets 8(3):184–194. 10.2174/187152709788680706 19601816 10.2174/187152709788680706

[4] Cao Y, Langer R, Ferrara N (2023) Targeting angiogenesis in oncology, ophthalmology and beyond. Nat Rev Drug Discov 22(6):476–495. 10.1038/s41573-023-00671-z 37041221 10.1038/s41573-023-00671-z

[5] Ferrara N, Hillan KJ, Gerber HP, Novotny W (2004) Discovery and development of bevacizumab, an anti-VEGF antibody for treating cancer. Nat Rev Drug Discov 3(5):391–400. 10.1038/nrd1381 15136787 10.1038/nrd1381

[6] Gerstner ER, Batchelor TT (2012) Antiangiogenic therapy for glioblastoma. Cancer J 18(1):45–50. 10.1097/PPO.0b013e3182431c6f 22290257 10.1097/PPO.0b013e3182431c6f PMC3269655

[7] Jo J, Wen PY (2018) Antiangiogenic therapy of High-Grade gliomas. Prog Neurol Surg 31:180–199. 10.1159/000467379 29393186 10.1159/000467379

[8] Friedman HS, Prados MD, Wen PY et al (2009) Bevacizumab alone and in combination with Irinotecan in recurrent glioblastoma. J Clin Oncol 27(28):4733–4740. 10.1200/JCO.2008.19.8721 19720927 10.1200/JCO.2008.19.8721

[9] Moen MD (2010) Bevacizumab: in previously treated glioblastoma. Drugs 70(2):181–189. 10.2165/11203890-000000000-00000 20108991 10.2165/11203890-000000000-00000

[10] Balañá C, Etxaniz O, Bugés C, Martínez A (2011) Approval denied by the European medicines agency (EMA) for bevacizumab in the treatment of high-grade glioma recurrence: a good Idea or a grave error? Clin Transl Oncol 13(3):209–210. 10.1007/s12094-011-0642-9 21421467 10.1007/s12094-011-0642-9

[11] Kreisl TN, Kim L, Moore K et al (2009) Phase II trial of single-agent bevacizumab followed by bevacizumab plus Irinotecan at tumor progression in recurrent glioblastoma. J Clin Oncol 27(5):740–745. 10.1200/JCO.2008.16.3055 19114704 10.1200/JCO.2008.16.3055 PMC2645088

[12] Raizer JJ, Grimm S, Chamberlain MC et al (2010) A phase 2 trial of single-agent bevacizumab given in an every-3-week schedule for patients with recurrent high-grade gliomas. Cancer 116(22):5297–5305. 10.1002/cncr.25462 20665891 10.1002/cncr.25462

[13] Brandes AA, Finocchiaro G, Zagonel V et al (2016) AVAREG: a phase II, randomized, noncomparative study of Fotemustine or bevacizumab for patients with recurrent glioblastoma. Neuro Oncol 18(9):1304–1312. 10.1093/neuonc/now035 26951379 10.1093/neuonc/now035 PMC4998997

[14] Vredenburgh JJ, Desjardins A, Herndon JE et al (2007) Bevacizumab plus Irinotecan in recurrent glioblastoma multiforme. J Clin Oncol 25(30):4722–4729. 10.1200/JCO.2007.12.2440 17947719 10.1200/JCO.2007.12.2440

[15] Taal W, Oosterkamp HM, Walenkamp AME et al (2014) Single-agent bevacizumab or lomustine versus a combination of bevacizumab plus lomustine in patients with recurrent glioblastoma (BELOB trial): a randomised controlled phase 2 trial. Lancet Oncol 15(9):943–953. 10.1016/S1470-2045(14)70314-6 25035291 10.1016/S1470-2045(14)70314-6

[16] Field KM, Simes J, Nowak AK et al (2015) Randomized phase 2 study of carboplatin and bevacizumab in recurrent glioblastoma. Neuro Oncol 17(11):1504–1513. 10.1093/neuonc/nov104 26130744 10.1093/neuonc/nov104 PMC4648304

[17] Weathers SP, Han X, Liu DD et al (2016) A randomized phase II trial of standard dose bevacizumab versus low dose bevacizumab plus lomustine (CCNU) in adults with recurrent glioblastoma. J Neurooncol 129(3):487–494. 10.1007/s11060-016-2195-9 27406589 10.1007/s11060-016-2195-9 PMC5021605

[18] Chamberlain MC, Johnston SK (2010) Salvage therapy with single agent bevacizumab for recurrent glioblastoma. J Neurooncol 96(2):259–269. 10.1007/s11060-009-9957-6 19593660 10.1007/s11060-009-9957-6

[19] Chen Y, Guo L, Li X, Liu R, Ren C, Du S (2020) Reduced-dose bevacizumab vs. standard-dose bevacizumab in recurrent high-grade glioma: which one is better? A meta-analysis. Clin Neurol Neurosurg 198:106239. 10.1016/j.clineuro.2020.106239 33007724 10.1016/j.clineuro.2020.106239

[20] Blumenthal DT, Mendel L, Bokstein F (2016) The optimal regimen of bevacizumab for recurrent glioblastoma: does dose matter? J Neurooncol 127(3):493–502. 10.1007/s11060-015-2025-5 26721244 10.1007/s11060-015-2025-5

[21] Levin VA, Mendelssohn ND, Chan J et al (2015) Impact of bevacizumab administered dose on overall survival of patients with progressive glioblastoma. J Neurooncol 122(1):145–150. 10.1007/s11060-014-1693-x 25575937 10.1007/s11060-014-1693-x

[22] Ajlan A, Thomas P, Albakr A, Nagpal S, Recht L (2017) Optimizing bevacizumab dosing in glioblastoma: less is more. J Neurooncol 135(1):99–105. 10.1007/s11060-017-2553-2 28667595 10.1007/s11060-017-2553-2

[23] Wen PY, Chang SM, Van den Bent MJ, Vogelbaum MA, Macdonald DR, Lee EQ (2017) Response assessment in Neuro-Oncology clinical trials. J Clin Oncol 35(21):2439–2449. 10.1200/JCO.2017.72.7511 28640707 10.1200/JCO.2017.72.7511 PMC5516482

[24] Common Terminology (2017)

[25] Louis DN, Perry A, Wesseling P et al (2021) The 2021 WHO classification of tumors of the central nervous system: a summary. Neuro Oncol 23(8):1231–1251. 10.1093/neuonc/noab106 34185076 10.1093/neuonc/noab106 PMC8328013

[26] R Core Team A Language and Environment for Statistical Computing. R Foundation for Statistical Computing, Vienna, Austria. Published online 2024. https://www.R-project.org/

[27] Cohen MH, Shen YL, Keegan P, Pazdur R (2009) FDA drug approval summary: bevacizumab (Avastin) as treatment of recurrent glioblastoma multiforme. Oncologist 14(11):1131–1138. 10.1634/theoncologist.2009-0121 19897538 10.1634/theoncologist.2009-0121

[28] Franke AJ, Skelton WP, Woody LE et al (2018) Role of bevacizumab for treatment-refractory meningiomas: A systematic analysis and literature review. Surg Neurol Int 9:133. 10.4103/sni.sni_264_17 30090665 10.4103/sni.sni_264_17 PMC6057170

[29] Gleeson JP, Keane F, Keegan NM et al (2020) Similar overall survival with reduced vs. standard dose bevacizumab monotherapy in progressive glioblastoma. Cancer Med 9(2):469–475. 10.1002/cam4.2616 31756059 10.1002/cam4.2616 PMC6970030

[30] Melhem JM, Tahir A, Calabrese E et al (2023) Dose-dependent efficacy of bevacizumab in recurrent glioblastoma. J Neurooncol 161(3):633–641. 10.1007/s11060-023-04248-z 36749445 10.1007/s11060-023-04248-z

[31] Youssef G, Rahman R, Bay C et al (2023) Evaluation of standard response assessment in Neuro-Oncology, modified response assessment in Neuro-Oncology, and immunotherapy response assessment in Neuro-Oncology in newly diagnosed and recurrent glioblastoma. J Clin Oncol 41(17):3160–3171. 10.1200/JCO.22.01579 37027809 10.1200/JCO.22.01579

[32] Wen PY, van den Bent M, Youssef G et al (2023) RANO 2.0: update to the response assessment in Neuro-Oncology criteria for High- and Low-Grade gliomas in adults. J Clin Oncol 41(33):5187–5199. 10.1200/JCO.23.01059 37774317 10.1200/JCO.23.01059 PMC10860967

[33] Motoo N, Hayashi Y, Shimizu A, Ura M, Nishikawa R (2019) Safety and effectiveness of bevacizumab in Japanese patients with malignant glioma: a post-marketing surveillance study. Jpn J Clin Oncol 49(11):1016–1023. 10.1093/jjco/hyz125 31665343 10.1093/jjco/hyz125 PMC6923818

[34] Sirven-Villaros L, Bourg V, Suissa L et al (2018) Bevacizumab: is the lower the better for glioblastoma patients in progression? Bull Cancer 105(12):1135–1146. 10.1016/j.bulcan.2018.07.010 30301554 10.1016/j.bulcan.2018.07.010

[35] Vredenburgh JJ, Cloughesy T, Samant M et al (2010) Corticosteroid use in patients with glioblastoma at first or second relapse treated with bevacizumab in the BRAIN study. Oncologist 15(12):1329–1334. 10.1634/theoncologist.2010-0105 21147867 10.1634/theoncologist.2010-0105 PMC3227925

[36] Salvalaggio A, Pini L, Gaiola M et al (2023) White matter tract density index prediction model of overall survival in glioblastoma. JAMA Neurol 80(11):1222–1231. 10.1001/jamaneurol.2023.3284 37747720 10.1001/jamaneurol.2023.3284 PMC10520843

[37] Boxerman JL, Snyder BS, Barboriak DP, Schmainda KM (2023) Early post-bevacizumab change in rCBV from DSC-MRI identifies pseudoresponse in recurrent glioblastoma: results from ACRIN 6677/RTOG 0625. Front Oncol 13:1061502. 10.3389/fonc.2023.1061502 36776298 10.3389/fonc.2023.1061502 PMC9909012

[38] Schmainda KM, Zhang Z, Prah M et al (2015) Dynamic susceptibility contrast MRI measures of relative cerebral blood volume as a prognostic marker for overall survival in recurrent glioblastoma: results from the ACRIN 6677/RTOG 0625 multicenter trial. Neuro Oncol 17(8):1148–1156. 10.1093/neuonc/nou364 25646027 10.1093/neuonc/nou364 PMC4490871

