# Supplementary material for: Bevacizumab in recurrent glioblastoma: does dose matter? Our monocentric and comparative experience
Source: J Neurooncol. 2025 Mar 10;173(2):449–56. doi: 10.1007/s11060-025-04992-4 (PMC12106581; doi:10.1007/s11060-025-04992-4)
Supplement: Supplementary file 1 — Supplementary Material 1 [file 11060_2025_4992_MOESM1_ESM.docx]

**Supplementary materials**

**Table 1S.** Response rates according to RANO criteria in patients treated with 5 or 10 mg/kg bevacizumab for recurrent glioblastoma. Seven patients could not be assessed for response due to death, progressive disease in the first cycle, or failure to reach the first follow-up MRI scan.

| Response | Patients treated with 5 mg/Kg bevacizumab (n=28) | Patients treated 10 mg/Kg bevacizumab (n=46) | p-value |
| --- | --- | --- | --- |
| Complete response  Partial response  Stable disease  Progression of disease | 0 (0%)  7 (25%)  5 (18%)  16 (57%) | 0 (0%)  6 (13%)  19 (41%)  21 (46%) | 0.07 |
| Objective response rate | 7 (25%) | 6 (13%) | 0.32 |
| Disease control rate | 12 (43%) | 25 (54%) | 0.47 |

**Table 2S**. Summary of studies with bevacizumab as monotherapy for recurrent glioblastoma

| **Study** | **Type of trial** | **N° patients** | **BEV dose/schedule** | **PFS (months)** | **OS (months)** | **Toxicity (% patients grade ≥3 AEs)** | **Decreased steroid use (% of patients)** |
| --- | --- | --- | --- | --- | --- | --- | --- |
| Kreisl, 2009 (NCI 06-C-0064E) ^12^ | Phase II | 48 | BEV 10 mg/Kg q2w | mPFS 4  PFS6 29% | mOS 4.75 | 27% | 58% |
| Raizer, 2010 ^13^ | Phase II | 50 | BEV 15 mg/kg q3w | PFS6 25% | mOS 6.5 | 18% | / |
| Brandes (AVAREG trial), 2016 ^14^ | Phase II | 91 | BEV 10 mg/kg q2w vs fotemustine (FTM) 75 mg/m^2^ on days 1, 8, and 15, then 100 mg/m^2^ q3w | mPFS BEV 3.38  (mPFS FTM 3.45) | mOS BEV 7.3  (mOS FTM 8.7) | BEV 17%  (FTM 47%) | 23 % (BEV arm) |
| Reardon (CheckMate143), 2020^40^ | Phase III | 185 | BEV 10 mg/Kg q2w or nivolumab 3 mg/Kg q2w | mPFS BEV 3.5  mPFS nivolumab 1.5 | mOS BEV 10  mOS nivolumab 9.8 | 15.2% BEV  18.1% nivolumab | / |
| Chamberlain, 2010 ^19^ | Retrospective | 50 | BEV 10 mg/Kg q2w | mPFS 10  PFS6 42% | mOS 8.5 | 18% | 33% |
| Gleeson, 2020 ^32^ | Retrospective | 118 | BEV 10 mg/Kg q2w vs BEV 5 mg/Kg q2w or BEV 7.5 mg/Kg q3w | / | mOS 5.8 (no difference between doses p-value 0.584) | / | 16.8% SD  11.2% LD |

**Table 3S**. Summary of studies of bevacizumab in combination with cytotoxic agents (irinotecan, fotemustine, lomustine, temozolomide) in recurrent glioblastoma

| **Trial** | **Type of trial** | **N° patients** | **Dose/schedule** | **PFS** | **OS** | **Toxicity (% patients grade ≥ 3 AEs)** | **Steroid use** |
| --- | --- | --- | --- | --- | --- | --- | --- |
| Vredenburgh, 2007 ^15^ | Phase II | 23+12 | Cohort 1: BEV 10 mg/kg q2w+ IRI 125-340 mg/m^2^ q2w  Cohort 2: BEV 15 mg/kg q3w + IRI on days 1, 8, 22, and 29 of q6w, 350 mg/m^2^ or 125 mg/m^2^ | mPFS 6 months | mOS 10.5 months | 31.5% treatment interruption | / |
| Friedman (BRAIN study), 2009 ^9^ | Phase II | 167 | BEV 10 mg/kg q2w alone vs in combination with irinotecan (IRI) 125-340 mg/m^2^ | 6 months PFS (BEV alone vs BEV+IRI) 42.6% and 50.3% respectively | mOS BEV 9.2  mOS BEV+IRI 8.7 months | 46.4% (BEV) and 65.8% (comb) | No steroid reduction detected |
| Reardon, 2011 | Phase II | 40 | Carboplatin AUC 4 mg/ml-min day1 + BEV 10 mg/kg + irinotecan 340-125 mg/m2 day 1 and 14 | mPFS 5.9 | mOS 8.3 | 68% | / |
| Desjardins, 2012^41^ | Phase II | 32 | TEM 50 mg/m2 daily + BEV 10 mg/kg q2w | mPFS 4 | mOS 9.2 | 25% | 31% |
| Taal (BELOB trial), 2014 ^16^ | Phase II | 153 | Lomustine (LOM) 110 mg/m^2^ q6w vs BEV 10 mg/kg q2w vs combination treatment LOM + BEV | mPFS (BEV, LOM, BEV+LOM respectively)  3, 1, 4 months | mOS  8, 8, 12 months | 44% BEV  61% LOM  71%  BEV+LOM | / |
| Soffietti, 2014 ^42^ | Phase II | 54 | BEV 10 mg/kg q2w + FTM 75 mg/m2 day 1 and day 8, followed by maintainance phase | mPFS 5.2 | mOS 9.1 | 37.3% | / |
| Sepùlveda, 2015 ^43^ | Phase II | 32 | BEV 10 mg/kg q2w + TMZ 150 mg/m2 days 1–7 and 15–21 q4w | mPFS 4.2 | mOS 7.3 | 50% | / |
| Field, 2015 ^17^ | Phase II | 122 | BEV 10 mg/kg q2w + carboplatin every 4 weeks (AUC 5)  vs BEV monotherapy | mPFS 3.5 months (for both arms) | mOS (combo) 6.9 months mOS (BEV) 7.5 months | 64% in combination arm vs 58% in BEV alone arm | / |
| Weathers, 2016 ^18^ | Phase II | 71 | BEV monotherapy (10 mg/kg) vs low dose BEV (5 mg/kg) + LOM (90 mg/m2) | In patients with GBM first recurrence, mPFS in the low dose BEV + LOM was 4.96 months, CI: 4.17-13.44  In the BEV alone arm it was 3.22 months CI: 2.5-6.01, p = 0.08 | mOS in patients with first GBM recurrence on low dose BEV + LOM was 13.05 months (95% CI: 7.08-17.82) vs BEV alone 8.8 months, CI: 6.42-20.22, p =0.98 | 24 grade ≥ 3 adverse events in combination arm vs 18 in BEV alone arm | / |
| Badruddoja, 2018^44^ | Phase II | 30 | BEV 10 mg/kg q2w + temozolomide 100 mg/mq days 1–5 and days 15–19 q4w | mPFS 5.6 | mOS 12.4 | 37% | / |
| Reyes-Botero, 2018 ^45^ | Phase II | 66 | TMZ 130–150 mg/m2 per day for 5 days q4w + BEV 10 mg/kg q2w | mPFS 3.8 | mOS 6 | 54% | / |
| Wick, 2017 ^46^ | Phase III | 437 | Cohort 1: lomustine 90 mg/sm q6w + BEV 10 mg/Kg q2w  Cohort 2: lomustine 110 mg/sm | mPFS 4.2  mPFS 1.5 | mOS 9.1 (comb.)  mOS 8.6 (monotp) | 63.6% comb arm  38.1% monotherapy | 61.1% stable/reduced  61.5% stable/reduced |
| Huang, 2020 ^47^ | Retrospective | 22 | BEV 10 mg/kg q2w, 1 mg/m2 vincristine, and 300 mg/m2 carboplatin | mOS 10 | mPFS 4.5 | / | / |
